# Incidental Emotions and Cooperation in a Public Goods Game

**DOI:** 10.3389/fpsyg.2022.800701

**Published:** 2022-03-10

**Authors:** Yen Nguyen, Charles N. Noussair

**Affiliations:** ^1^Department of Economics, Tilburg University, Tilburg, Netherlands; ^2^Department of Economics, University of Arizona, Tucson, AZ, United States

**Keywords:** emotion, cooperation, virtual reality, experiment, free-riding

## Abstract

The study reported here considers the relationship between emotional state and cooperation. An experiment is conducted in which the emotions of fear, happiness, and disgust are induced using 360-degree videos, shown in virtual reality. There is also a control condition in which a neutral state is induced. Under the Fear, Happiness, and Disgust conditions, the cooperation level is lower than under the Neutral condition. Furthermore, cooperation declines over time in the three emotion conditions, while it does not under Neutral. The findings suggest that emotions are associated with the dynamic pattern of declining cooperation over time.

## Introduction

Cooperation is the sacrifice of one’s individual interest to increase social welfare. Cataloging the determinants of cooperative behavior has attracted a great deal of interest from economists and other social scientists. Experimental research has established that the level of cooperation follows predictable patterns, and numerous correlates of cooperative behavior have been identified. Nonetheless, among individuals, there is considerable heterogeneity in the propensity to cooperate. Indeed, the same individual may cooperate in one instance, and then shortly thereafter, in a similar situation, behave totally selfishly. One potential source of this variability is the decision maker’s emotional state, which differs across individuals and changes over time, sometimes rapidly. In traditional theories of economic decision-making, the role of emotions has typically been neglected. The link between emotional state and the tendency to cooperate is the topic of the study reported here*.

One of the most widely used experimental paradigms to investigate the circumstances under which individuals cooperate is the Voluntary Contributions Mechanism (VCM). Originally studied in a somewhat different form by Marwell and Ames (1979) and Dawes (1980), this paradigm is also often referred to as the Public Good game. In this game, a number of agents in a group each have an endowment, which each agent can allocate, in any proportion, between a private and a group account. The amount that an individual puts into her private account is hers to keep. The amount placed into the group account is multiplied by a factor greater than 1 (though lower than the number of players, N) by the experimenter, and the resulting total is divided equally among all group members. These incentives mean that each individual has a dominant strategy to place the entirety of her endowment into the private account, while the strategy profile that maximizes the group’s total payoff is for all players to place their whole endowment into the group account. The amount placed into the group account is referred to as a contribution, and the percentage of endowment contributed is taken as a measure of cooperation. Thus, the VCM paradigm permits measurement and comparison, both between individuals and groups, of the extent of self- versus group-interested behavior.

It was established early on that cooperation is not uncommon but also not universal (Dawes, 1980). With repetition of the game, cooperation declines (Andreoni, 1988; Isaac and Walker, 1988a). There are a number of correlates of cooperation, most prominently the marginal-per-capita return (Isaac and Walker, 1988a), the amount that each unit contributed to the group account yields to each group member (the higher the marginal-per-capita-return, the more that is contributed to the group account). Changes to the institutional structure, such as permitting communication (Isaac and Walker, 1988b), as well as allowing for peer-to-peer punishment (Yamagishi, 1986; Ostrom et al., 1992; Fehr and Gächter, 2000) also can increase cooperation.

The characteristics of participants can also influence the level of cooperation that a group exhibits. Some correlates include program of study (Marwell and Ames, 1981), risk attitude (Teyssier, 2012; Kocher et al., 2015), and level of cognitive sophistication (Lohse, 2016)^1^. The level of cooperation is also influenced by extent to which players have preferences to reciprocate kind or unkind actions. There is strong empirical evidence of a correlation between cooperation in the public goods game and expectations about the cooperation of others (Fischbacher et al., 2001; Fischbacher and Gaechter, 2010; Bechtel and Scheve, 2017).

Some work has considered the correlation between personality and cooperation. Balliet et al. (2009) conduct a meta-analysis of studies relating Social Value Orientation (SVO, Messick and McClintock, 1968) to cooperativeness. They find that the SVO measure correlates with cooperation level, with less competitive individuals cooperating more. Hilbig and Zettler (2009) show that those exhibiting greater values of the personality dimension of honesty-humility are more cooperative. Thielmann et al. (2020) find, among other results, that agreeableness and environmentalism correlate positively with cooperation in a social dilemma.

Though less explored, it is quite plausible that transitory forces affecting participants at the time their decisions are made could matter as well. Here, we consider whether the emotional state of participants is a determinant of behavior. We conduct an experiment in which we induce, in different treatments, three emotional states: happiness, fear, and disgust, as well as a neutral state that serves as a control treatment. We then compare the resulting level and dynamics of cooperation under the different emotional states. We induce, rather than track, emotional state, in order to be able to establish causal relationships between emotional state and cooperation.

Indeed, many psychologists view emotions as a key determinant of human cooperation, and assert that cooperative behavior is affected differently by different emotions (Fessler et al., 2015). Several different mechanisms have been proposed. The *Affect Infusion Model* (Forgas, 1995, 2001) argues that emotional state colors one’s decision-making process, so that, for example, a positive emotional state might affect beliefs about the likelihood that outcomes will be positive or negative. The *Affect as Information* framework (Schwarz and Clore, 1988, 2003) posits that one’s emotional state is used as an informative input into the decision process, e.g., if one is in a fearful state, it is interpreted as a sign that there is adverse risk possible in the decision one is making, and that one should avoid the risk.

In experimental economics, the connection between emotions and cooperation has been explored by a number of authors. Drouvelis and Grosskopf (2016) show that cooperation is sensitive to subjects’ current emotional state. Specifically, a happy emotional state leads to higher contributions, and an angry state leads to lower contributions, to a public good. In a similar vein, Joffily et al. (2014) report that a more positive emotional state is associated with greater cooperation. Boyce et al. (2016) find that sadness or happiness does not affect the willingness-to-pay for environmental goods. Capra (2004) observes that a positive emotional state increases giving in dictator games. These studies build on a long tradition in management and psychology studying emotions and cooperation. For example, Hertel and Fiedler (1994) consider the effect of positive and negative emotional states, induced using film clips, on cooperation framed as an airplane maintenance task. They find that their mood induction did not affect the average level of cooperation, but they did observe that positive mood increased the variability of cooperation. Other studies investigate cooperative behavior in response to shame and guilt (de Hooge et al., 2007), gratitude (DeSteno et al., 2010), and anger (Motro et al., 2016)^2^.

Clarifying the relationship between emotional state and cooperation can shed light on the ongoing debate about whether cooperation is intuitive. Rand et al. (2012) report that cooperative behavior is intuitive. They use time pressure to elicit spontaneous behavior, and they observe that decisions taken under time pressure tend to be more cooperative^3^. However, Kvarven et al. (2020), in a meta-analysis of 82 studies on intuition and cooperation, show that the relation between intuition and cooperation is driven by six studies in which the use of emotional processing was manipulated. For example, Levine et al. (2018) report that, when informed that players in a prisoners’ dilemma used emotion to determine their action, observers thought that the players were more likely to have cooperated. Players who reported using emotion rather than reason in their own decisions, as well as those who thought their partner employed emotion, were also more likely to cooperate. Participants instructed to use emotion in their decisions were more likely to cooperate. Gärtner et al. (2022) find that inducing an affective decision mode increased pro-social behavior in five of the six paradigms they studied, including the prisoners’ dilemma, but the exception was the Public Good Game. These results indicate that a deeper understanding of the relation between emotional state and cooperation is needed. In particular, isolating the effect of specific emotions such as happiness, fear and disgust on cooperation, may clarify the precise manner whereby emotional processing and cooperation are associated.

In our experiment, we induce three different emotional states and a neutral state, and then observe behavior in a repeated Public Good game. The conditions are Fear, Happiness, Disgust, and a Neutral treatment. A positive relationship between happiness and cooperation has been documented by Drouvelis and Grosskopf (2016) and thus our evaluation of the effect of happiness represents a conceptual replication of this earlier result. To our knowledge, the effect of fear and disgust on cooperation have not been studied. Each of the three emotions are among the six basic universal emotions as cataloged by Ekman and Friesen (1975). We find that Fear, Happiness, and Disgust all result in lower contributions compared to the Neutral treatment. In other words, the incidental emotions we study, whether positive or negative in valence, result in less cooperation than occurs under the Neutral treatment.

Our approach is novel in terms of method. In particular, to induce emotional states, we employ a new research tool, the use of immersive 360-degree videos shown in virtual reality. One commonly used traditional means of emotion induction is the use of film clips shown on a computer screen. It has been argued that the use of film clips as emotion-inducing stimuli is advantageous compared to showing still pictures, since the dynamic nature of films creates more realism (Dhaka and Kashyap, 2017). Film clips are typically regarded as an effective mood induction method (Westermann et al., 1996). A major advantage of film clips is that they can be used without explicit instructions that can tip participants off about the fact that the experimenter intends to induce a certain emotional state (Kuijsters et al., 2016).

Gomez et al. (2009) assess the persistence of different moods induced by film clips during a computerized task. They find that emotion induction via film clips still lasted after nine minutes. After that time interval, participants who had a negative emotional state induction report more negative emotional valence than those who had a positive induction. The results also suggest that induced changes in positive and negative emotional states are maintained throughout an intervening task. Murray et al. (1990), also found that neutral and positive moods induced with film clips were sustained after an intervening cognitive task on categorization of about 9 min. The effects of audio-visual emotion induction are presumably further reinforced when using 360-degree videos shown in virtual reality. Hence, we posit that the emotion induction via VR would last considerably longer than 9 min.

The use of virtual reality is potentially particularly valuable in inducing negative withdrawal emotions such as fear or disgust. This is because it is difficult to guarantee that individuals’ attention is on aversive videos when they are shown in a conventional manner on computer screens, since it is possible to avert one’s gaze. Looking away from the stimulus is not possible in a 360-degree video, in which the video appears in every direction^4^. The videos are shown with individually head-mounted Oculus Rift™ gear to display 360-degree videos to subjects. Such videos create a fully immersive environment while simultaneously giving users full control of their angle of view in the pre-recorded footage^5^. Subjects are completely and inescapably surrounded by the audio-visual stimuli, minimizing their awareness of being in a physical laboratory environment. The video is filmed from the point of view of a participant in the video, rather than that of an observer. As a result, virtual reality presumably creates more powerful emotion induction than conventional techniques. The procedures were approved by the Institutional Review Board of the University of Arizona, and a representative of the IRB viewed each video prior to its use in any study.

The balance of the prior evidence is that positive emotional states are associated with more cooperation and negative emotions with more self-interested behavior. One possible mechanism for this effect is a preference for conditional cooperation (Fischbacher et al., 2001) coupled with the Affective Generalization Hypothesis proposed by Johnson and Tversky (1983). Under the Affective Generalization Hypothesis, positive emotional states lead to more optimistic beliefs, while negative states lead to pessimism. Thus, if one would like to cooperate only if others cooperate as well, a positive mood might make one have stronger beliefs that others will cooperate. This makes one more likely to cooperate as well. Similarly, one of the negative emotional states would make an individual less likely to cooperate than under a Neutral condition, by inducing more pessimistic beliefs. This hypothesized effect of happiness is line with the study of Drouvelis and Grosskopf (2016), who find that happiness leads to more cooperation, and the effect of negative emotions is consistent with Motro et al. (2016), who find that anger reduces cooperation. This account is plausible to us, and thus we posit that an emotion with positive valence, happiness, will result in higher contributions than the Neutral condition. We also hypothesize that the emotions with negative valence, fear and disgust, will result in lower contributions than the Neutral condition. Because the hypothesis is consistent with prior work, it can be viewed as a replication hypothesis, with the replication conceptual since we depart considerably from the procedures of the earlier studies.

**Hypothesis 1:** Happiness will result in higher contributions than the Neutral condition, while Fear and Disgust will result in lower contributions than the Neutral condition.

Prior studies typically find that contributions decay over time (Andreoni, 1988; Isaac and Walker, 1988a; for a review see Chaudhuri, 2011). However, this prior work has not controlled for or induced emotional states. Thus, while it is not evident that the decline would be observed in each of our conditions, in the absence of any contradictory evidence, we hypothesize that:

**Hypothesis 2:** Contributions decrease over time in all treatments.

This paper is structured as follows. Section 2 describes the experiment and presents the hypotheses. Section 3 reports the results and Section 4 contains a brief discussion.

## Experimental Design

All sessions of the experiment were conducted at the Economic Science Laboratory, located at Eller College of Management, University of Arizona, Tucson, Arizona, United States, in early 2018. All 141 participants in the study were University of Arizona undergraduate students, who self-enrolled for the experiment through the recruitment system of the laboratory. All participants were between 18 and 25 years old. The experiment was computerized using the Z-tree software package (Fischbacher, 2007) and conducted in English. The groups playing the game always consisted of either three or four participants. There was only one group participating in each session, due to the fact that the laboratory only had 4 VR headsets available^6^. There were 17, 19, 21, and 18 women in the Neutral, Happiness, Fear and Disgust treatments, respectively. There were 18, 17, 14, and 17 male participants in the four treatments.

The sample size was chosen based on calculations of statistical power. Our sample sizes in each treatment allow us to detect a medium-sized effect of d = 0.5 (Cohen, 1988) with a probability of at least of.665 if the hypothesis test of a treatment difference is one-sided. We conducted a sensitivity analysis using G-Power™ to calculate the power to detect an effect size of 0.5 between each pair of treatments given the sample size in each treatment, using a *t*-test for independent sample means and applying α < 0.05 as a standard of statistical significance. The power to detect an effect of d = 0.5 is 0.665 −0.670 depending on the pair of treatments being compared (the sample sizes in each treatment have slight differences). We have a power of 0.8 of detecting an effect of d = 0.596 −0.600, depending on the treatments being compared.

Virtual Reality technology (Oculus Rift headsets) was used to play the immersive 360-degree videos that were used for the emotion induction. The Economic Science Laboratory had previously conducted a validation study on the effectiveness of these particular videos in increasing the intended emotion without producing unintended emotions. The Neutral video was selected because it did not significantly increase the reported level of any emotion when it was viewed. See Medai and Noussair (2021) for the results for the happiness, fear and neutral videos and Kugler et al. (2020) for the disgust video^7^. On the bases of these earlier manipulation checks, the videos were chosen for emotion induction in this experiment. Neutrality was induced with a video of a field of flowers. Fear was induced with a video in which the subject is walking on a tightrope across a steep canyon. The happiness video was one in which the subject was surfing in the tropics, and disgust was created with a video of disgusting things found in food. Each video was played for 5 - 6 min. The experimental design was between-subject. Each individual had only one emotion induced and all individuals in a session knew that they are watching the same video. They viewed the video simultaneously.

After the experimenter read the instructions for the game aloud, subjects played ten periods of the Voluntary Contributions Mechanism^8^. The four members of each group interacted repeatedly and anonymously for 10 periods. The specific parameters were the following. In each period, each participant received an initial endowment of 20 tokens referred to as “Experimental Currency Units” (ECU; with a conversion rate of 17 ECU = 1 $US). Players then simultaneously decided how to allocate the 20 tokens. A participant could contribute any number of tokens to a “project,” which benefited all players equally and keep the remaining tokens for herself. The marginal per-capita return to the project equaled 0.5. In other words, each token contributed to the project yielded a payoff of 0.5 tokens to each of the four group members. Thus, if all players contributed their entire endowment to the project, each player would receive double the earnings that she would if they all contributed zero.

Specifically, the payoff function in each period was:


$$\pi_{i}=\left(20-c_{i}+0.5*\sum_{j=1}^{n}c_{j}\right),$$

where π_*i*_ is individual *i*’s payoff and *i*’s contribution to the project is denoted by *c*_*i*_. At the end of each period, participants were shown a summary screen that informed them of the sum of all contributions *c*_*j*_ to the project and their earnings for the period.

In the experiment, the game is finitely repeated. If the game is played once, the only Nash equilibrium is for all players to contribute zero. Thus, the only subgame perfect equilibrium of the 10-period finitely repeated game of our experiment is for all players to contribute zero in each of the ten periods, regardless of the history of play. As a result, each group member would earn 20 ECU (Experimental Currency Units) in each period. If each player would contribute her full endowment to the group project, the maximum feasible group payoff would be attained. In this case, each group member would earn 40 ECU each period. As indicated earlier, strong empirical evidence exists that individuals cooperate more than in the subgame perfect equilibrium, but also exhibit less than full cooperation. The level of cooperation declines over time.

At the end of each period, participants are informed of the group’s total contribution and their own earnings, and are reminded of their own contribution. They are not informed about the individual contributions or the earnings of other group members. No communication between participants was possible. All periods counted towards participants’ monetary payment. Earnings averaged $US15 per subject. The duration of the instructions was approximately 10 min followed by 5 minutes of play of the game. The data and all materials are available from the authors.

## Results

In this section, we present the results of our empirical investigation into whether emotional states influence an individual’s contributions in a repeated linear public goods game^9^. Hypothesis 1 asserted that the positive emotional state of Happiness would enhance cooperation relative to Neutrality, while the two negative states, Fear and Disgust, would have the effect of reducing contributions. Our first finding, however, is the existence of quite a different pattern.

**Result 1. There is no difference among treatments in the initial period. Inducing emotions has no statistically detectable effect on early game behavior. In the final rounds of the game, however, subjects in the Neutral condition contribute significantly more on average than subjects assigned to the Happy, Fear and Disgust conditions.**

**FIGURE 1:**
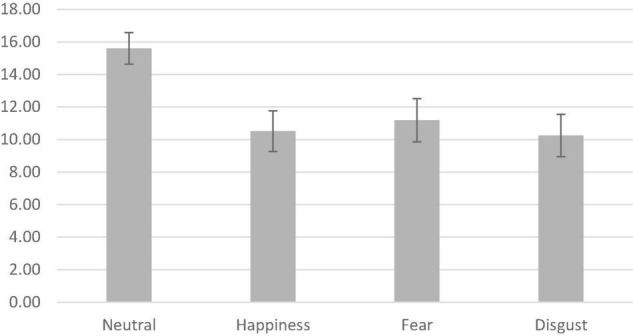
Average contribution, by treatment. The figure shows the average per-period contribution by treatment (Neutral, Happiness, Fear, Disgust), for the pooled data from all participants. The error bars are 95% confidence intervals for the means. The range of possible contributions is from 0 to 20. All periods and all participants are included.

Figure 1 depicts the average per period contribution by treatment (Neutral, Happiness, Fear, Disgust). The data shown in the figure are the average individual single-period contribution in ECU in each emotion treatment. The figure reveals the following patterns. The overall results indicate that the emotion treatments, Fear (9.7 tokens), Happiness (10 tokens), and Disgust (10.3 tokens), all exhibit lower contributions in comparison to the Neutral condition (12.7 tokens). Subjects in the Neutral treatment contribute an average of 27.5% more than under the three emotion treatments.

Table 1 considers whether the differences between treatments, in terms of average contribution, are significant. It reports the results from *t*-tests, conducted to determine whether the emotion treatments exhibit average contributions that are significantly different from each other. The tests are performed for the data from the first period, the last period, and for the ten periods overall.

**TABLE 1:** Results of *t*-tests of pairwise differences between treatments, for Period 1, Period 10, and all Periods 1 - 10.

Emotion treatments	Period 1	Average Period 1 – Period 10	Period 10
Neutral and Disgust	0.789	0.249	0.032**
Neutral and Fear	0.289	0.111	0.009***
Neutral and Happiness	0.829	0.381	0.045**
Happiness and Disgust	0.954	0.856	0.850
Happiness and Fear	0.420	0.569	0.593
Fear and Disgust	0.379	0.649	0.748

This table shows the results of t- tests that were conducted to evaluate the impact of the treatment on average contributions. The entries in the table are t-statistics. ***p < 0.01; **p < 0.05; *p < 0.1. Significance levels are Bonferroni uncorrected.

Table 1 reveals a number of interesting patterns. Hypothesis 1 asserted that the emotions with negative valence, fear and disgust, would lead to lower contributions compared to the Neutral condition. Conversely, the emotion with a positive valence, happiness, would lead to higher contributions compared to neutrality. The tests reported in the table indicate no treatment differences at the outset of play or for the ten periods considered as a whole. However, by period 10, there is significantly lower cooperation in the three emotion treatments than in the Neutral condition, while the three emotion treatments do not differ from each other.

We next consider whether the decay of contributions with repetition of the game appears under each of our emotion conditions, as proposed in Hypothesis 2. Our findings are stated as Result 2.

**Result 2. Contributions decline over time in the three emotion treatments, but not in the Neutral treatment.**

**FIGURE 2:**
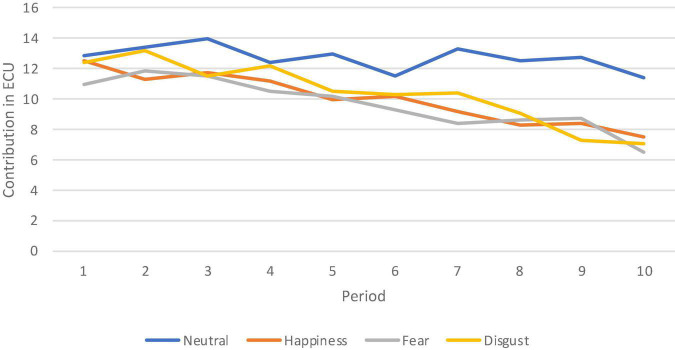
Average contribution in each period, by treatment. The figure shows the average contribution made in each period in each of the four treatments (Neutral, Happiness, Fear, Disgust).

Figure 2 below shows the average contribution made in each period in each of the four treatments. The data are averaged over all participants, separately for each treatment in which a given induced emotion was in effect. The average contribution in ECU, by period, is given on the vertical axis, and the period number is indicated on the horizontal axis for each treatment separately. The data in the three emotion treatments exhibit the following patterns. The average initial contributions are substantial, starting with a contribution of between 10 to 13 ECU out of a maximum of 20 in the first period, but decline as the game is repeated. A second pattern is that subjects contribute more in the Neutral treatment than in all three emotion treatments throughout the ten-period horizon, with the gap increasing over time. Contributions in the three emotion treatments converge downward and remain similar to each other over time.

We now consider whether the declining time trend in contributions over time is significant by conducting signed rank tests. Table 2 contains the results of signed-rank tests that were also conducted to evaluate the impact of the treatment on the change in average contribution between periods 1 and 10. The tests examine whether the distributions in periods 1 and 10 are significantly different from each other, using the sign and the ranking of the absolute magnitude of the change in the average contribution of a group between periods 1 and 10. Each group is treated as an observation, and there are 9 groups per treatment.

**TABLE 2:** Results of signed-rank tests of the change in average contribution between Periods 1 and 10.

Emotion treatment	Z	Prob > | z|
Neutral	− 0.713	0.476
Happiness	− 2.255	0.024
Fear	− 1.779	0.075
Disgust	− 2.196	0.028

*This table shows the results of signed-rank tests that were conducted to evaluate whether the change in average contribution between periods 1 and 10 is significant in each treatment. P-values are not Bonferroni corrected.*

The data reveal several interesting findings. Hypothesis 2 asserted that contributions would decrease over time in all treatments. The results from the signed rank tests report some ambiguity in this regard. We find compelling evidence that cooperation declines over time for all three emotion treatments, but not for the Neutral treatment. For the Neutral treatment, we fail to reject the null hypothesis of no change. For the Happiness and Disgust treatments, we can reject the null hypothesis of no change over time at *p* < 0.05. For the Fear treatment, we reject the null hypothesis at *p* < 0.1. As we discuss later in Section 4, one possible, though speculative, explanation for why the change over time under Neutral is not significant is that the Neutral emotion induction suppresses subjects’ integral emotions. In other words, when inducing neutrality, we are suppressing the emotions that would occur naturally in response to activity and outcomes in the game.

The regression estimates displayed in Tables 3A,B confirm the patterns that we have discussed above. The estimates are from the estimation of models assuming a random effect for each individual and robust standard errors. The dependent variable is individual *i*’s contribution in period *t*. The independent variables are treatment dummies and a time trend. One of the two specifications includes gender as a regressor. The estimates show that relative to the Neutral treatment, which is the baseline category, the three emotion treatments yield lower contributions. The negative coefficient on the period variable indicates that contributions decline over time. The lack of significance on the gender variable indicates that neither men or women contributed systematically more or less than the other. The similarity of the significance levels and estimates under the two specifications shows that the inclusion of gender as a variable does not alter the effects of treatment and time period.

**TABLE 3A:** The effect of treatment and period on the contribution of individual i.

Effect	Estimate	SE	95% CI		P
			*LL*	*UL*	
Random effects					
Constant	17.233	1.094	15.088	19.377	< 0.01
Happiness	−5.456	1.479	−8.354	−2.557	< 0.01
Fear	−6.314	1.540	−9.332	−3.296	< 0.01
Disgust	−3.895	1.559	−6.951	−0.839	< 0.05
Period	−0.527	0.067	−0.658	−0.396	< 0.01

*Number of observations = 1,410, R^2^ = 0.139.*

**TABLE 3B:** The effect of treatment, period, and gender on the contribution of individual i.

Effect	Estimate	SE	95% CI		p
			*LL*	*UL*	
Random effects					
Constant	17.734	1.068	15.641	19.827	< 0.01
Happiness	−5.145	1.537	−8.158	−2.132	< 0.01
Fear	−5.924	1.598	−9.056	−2.792	< 0.01
Disgust	−3.823	1.524	−6.810	−0.836	< 0.05
Gender	−1.337	1.164	−3.618	0.944	> 0.1
Period	−0.527	0.067	−0.658	−0.396	< 0.01

*Number of observations = 1,410, R^2^ = 0.146.*

A number of authors have noted (see for example Fischbacher et al., 2001) that there are distinct types of players in the Voluntary Contributions Mechanism. To investigate the possible effects of emotions on the incidence of different behavioral types, we classify subjects into three types of players: free-riders, conditional cooperators, and altruists, according to how they respond to the prior contributions of other group members. Consider an estimated regression with the functional form:


$$c_{i}^{t}=\alpha +\beta \,c_{a\,v\,g}^{t-1}+\varepsilon_{i}^{t}$$

where $c_{i}^{t}$ is the contribution of Individual *i* in period *t*, and $c_{a\,v\,g}^{t-1}$ is the average contribution in the group in period *t* - 1.

Subjects are classified as ‘free-riders’ when their estimated α=0 and β=0. This means that they contribute zero regardless of the past behavior of others. Subjects are considered ‘conditional cooperators’ when their β > 0, since they contribute more, the more cooperative the rest of their group was in the immediately preceding period. They are considered “altruists” when their estimated α > 0 and β=0. Altruists^10^ contribute a positive amount that does not depend on the past decisions of others. Subjects who meet none of these criteria are grouped under a category called ‘Other’. Table 4 below reports the distribution of the behavioral types as a percentage of all participants in each treatment.^11^

**TABLE 4:** Classification of participants into behavioral types.

Behavioral type	Neutral	Happiness	Fear	Disgust
Free-riders	0%	5.6%	2.9%	5.7%
Conditional Cooperators	51.4%	58.3%	74.3%	65.7%
Altruists	34.3%	16.7%	2.9%	5.7%
Other	14.3%	19.4%	20%	22.9%
**Total observations**	35	36	35	35

*The table reports the distribution of behavioral types by treatment as percentages of the participants in the treatment. Subjects are classified as free-riders when α=0 and β=0. Subjects are considered conditional cooperators when β > 0, and altruists when α > 0 and β=0.*

In Public Good Games, it is commonly found that a plurality of participants behave as “conditional cooperators,” i.e., people who are willing to contribute more if others contribute more as well (Fischbacher et al., 2001; Chaudhuri and Paichayontvijit, 2006). Our results from Table 4 confirm these findings. Furthermore, our results indicate that compared to the emotion treatments, the Neutral treatment has a greater proportion of altruists, and this appears to be associated with the absence of a decline of contributions in that treatment. There are more altruists in the Happiness than in the Fear and Disgust conditions. The Fear condition has the most conditional cooperators. Remarkably, free riders are completely absent in the Neutral treatment.

## Discussion

In this study, we applied a new emotion induction methodology, 360-degree videos shown in Virtual Reality, to study a fundamental question in the social sciences. Does an individual’s emotional state, specifically happiness, disgust or fear, have an effect on the individual’s tendency to cooperate? This study is an example of how emerging technologies can create new ways of conducting research in experimental economics. Technologies such as Virtual Reality can serve as useful complementary tools to existing emotion analysis and induction methods. While no one study can be definitive, and our sample is relatively small, we draw two conclusions from our findings.

The first conclusion is that *incidental emotions, whether positive or negative in valence, result in lower contributions compared to a Neutral state*. Our results indicate that on average, subjects contributed 27.5% less in the three emotion treatments, Fear, Happiness and Disgust, than they did in the Neutral condition and the differences are significant in later periods. More than one third of the subjects were classified as altruistic in the Neutral condition, which was 2 to 12 times the number of altruists in the three emotion treatments. The part of Hypothesis 1 that is supported is that negative emotions, Fear and Disgust, decrease contributions. The other part of Hypothesis 1, that positive emotions increase contributions, is not supported. Of course, this is only one study and future studies will allow for refinements of the results. In particular, they may establish whether some of the effects of emotions on behavior are too small to be detected with the number of participants we have employed.

The second conclusion is that we confirm that contributions decrease over time in all of the induced emotion treatments, but that they do not do so in the Neutral treatment. Thus, while Hypothesis 2, that contributions would decrease over time, is mostly supported, there is an important exception. The fact that our Neutral treatment does not exhibit the typical empirical pattern observed in prior studies is interesting. This suggests that inducing a Neutral emotion is not the same thing as not inducing an emotion at all. Hence, we propose the following conjecture*: Emotions are linked to a decrease in contributions in the Public Good game, perhaps because they lead to reciprocation of the behavior of other players. The Neutral treatment attenuates the decrease in cooperation by suppressing these emotions.* This last statement is certainly speculative, and further work focused directly on this mechanism would be required to evaluate the validity of this conjecture.

The differences that we observe among treatments do not appear immediately but open up after several periods of play. Thus, the emotions do not affect initial behavior, but interact with the dynamics of play to produce different outcomes in the Neutral treatment. In a standard Public Good game with no emotion induction, cooperation begins at an intermediate level and then declines over time. This dynamic pattern is also present in our Fear, Disgust, and Happiness treatments. In the Neutral treatment, the dynamics are affected by the Neutrality induction. We have seen that in the Neutral treatment, we do not observe pure free-riding. If the decline in cooperation over time that is typically observed is due to conditional cooperators responding to free-riding by lowering their own contributions, the lack of free-riders in the Neutral treatment eliminates this dynamic that generates the declining time trend.

There have been many studies studying the effects of emotions by means of emotion induction and this work has produced numerous valuable findings to aid our understanding of the relation between emotions and economic behavior. See for example the surveys by Baumeister et al. (2009), Izard (2009) and Lerner et al. (2016). In our opinion, a line of research using *emotion suppression* would also be beneficial in uncovering the role of emotions in behavior. Prior research on emotions and decision making has not considered, to our knowledge, whether a neutral emotion induction has a different effect from no emotion induction at all. It is not clear to us after conducting this study that Neutrality is in any sense a default emotion. A future avenue for study would therefore be to further investigate the particular effects of Neutrality. What does Neutrality really do? Does it cause people to behave differently in different tasks than they would behave otherwise? When does it do so? In our view, such a line of inquiry promises to yield very valuable insights.

There are several limitations to our study. The session size was limited by the number of VR headsets that we had available. The level of anonymity, while lower than it would be in a larger session, was the same among the treatments. It was also similar to the level that would exist in some workplace settings, where individuals might know who the other group members are, but cannot observe their specific actions. We recognize, however, that there may be an interaction effect on behavior between a lack of anonymity and emotional state. However, this would be an equal concern for any level of anonymity, and it is possible that the relationship between emotions and cooperation could differ at other levels of anonymity. The size of the sample was modest, our study was not preregistered, and we do not correct for multiple hypothesis testing, so our study can be considered as an initial exploration. Another limitation is that, although the individuals who participated in the manipulation checks for the videos were drawn from the same subject pool, the checks were conducted at different times and on different individuals than those who participated in the main experiment. Future research is needed to confirm our results.

## Data Availability Statement

The raw data supporting the conclusions of this article will be made available by the authors, without undue reservation.

## Ethics Statement

This study involving human participants were reviewed and approved by University of Arizona Institutional Review Board. The patients/participants provided their written informed consent to participate in this study.

## Author Contributions

Both authors listed have made a substantial, direct, and intellectual contribution to the work, and approved it for publication.

## Conflict of Interest

The authors declare that the research was conducted in the absence of any commercial or financial relationships that could be construed as a potential conflict of interest.

## Publisher’s Note

All claims expressed in this article are solely those of the authors and do not necessarily represent those of their affiliated organizations, or those of the publisher, the editors and the reviewers. Any product that may be evaluated in this article, or claim that may be made by its manufacturer, is not guaranteed or endorsed by the publisher.

## Figures and Tables

**FIGURE B1:**
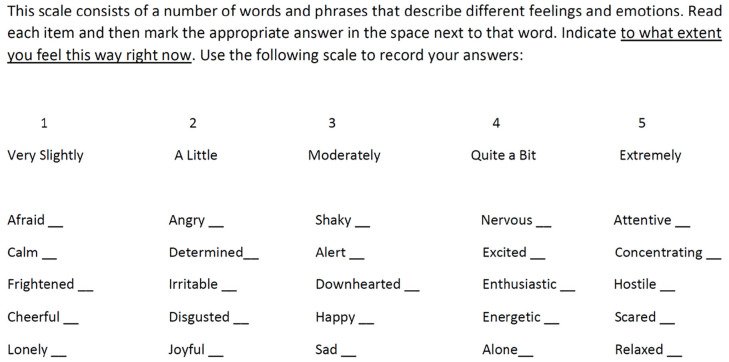
The questionnaire employed in the first two manipulation checks.

## Footnotes

1 Marwell and Ames (1981) find that students of economics tend to free-ride more than those in other programs of study. Kocher et al. (2015) find no relationship between risk aversion and contributions, while Teyssier (2012) observes that risk aversion and contributions are negatively correlated. Lohse (2016) documents a positive correlation between a cognitive ability measure, the Cognitive Reflection Test, and contribution level.

2 de Hooge et al. (2007) observe that guilt is associated with greater cooperation while shame is not. DeSteno et al. (2010) report that inducing gratitude on the part of players increases cooperation. Motro et al. (2016) find that under some conditions, anger reduces cooperation levels.

3 Tinghög et al. (2013) have called this result into question by showing that the relationship between time pressure and cooperativeness is only present if the data from those participants who did not make a decision within the allowable time frame are excluded.

4 Fear and disgust are among the emotions that have proven to be reliably induced using movies (Kreibig et al., 2007; Rottenberg et al., 2007).

5 Virtual reality has been previously employed in experimental economics to study trust (Kugler et al., 2020), the effect of peers on worker effort (Boensch et al., 2017), and the effect of being observed on honesty (Mol et al., 2020).

6 Most sessions had four participants, and our intention was to have exactly four participants in each session. On three occasions, only three individuals appeared at the sessions, and we proceeded to conduct the sessions with the three participants present with the same MPCR in effect. These data are included in the analysis. Previous studies report mixed results on whether larger groups are more or less cooperative given the same MPCR. Isaac et al. (1994), Carpenter (2007), Diederich et al. (2016) and Pereda et al. (2019) report that cooperation is greater for larger groups. On the other hand, Isaac and Isaac and Walker (1988a), Capraro and Barcelo (2015), Feltovich and Grossman (2015), and Nosenzo et al. (2015) report ambiguous results regarding the effect of group size on cooperation. Excluding the three person groups does not affect the results with regard to statistical significance, with the exception that the difference in contributions in period 10 between the Neutral and Disgust treatments is borderline significant at *p* = 0.054 rather than at *p* < 0.05.

7 In addition, we have recently (in late 2021) conducted two new manipulation checks of the Neutral, Fear and Happiness videos. These are reported in Appendix B, along with the results of a manipulation check for the Disgust video.

8 The experimenter carefully read the instructions to the participants. After the instructions, subjects answered control questions to test their understanding of the rules of the experiment. See Appendix A for the instructions and the control questions. After all subjects finished the control questions, the experimenter checked their answers and to ensure correct understanding, explained the correct answers to any questions answered incorrectly privately to the individual. Then, the computerized experiment was initiated through launching of the Z-tree program.

9 No treatments or observations are excluded from the analysis that we report here in this paper. All of the statistical tests that we have conducted are reported.

10 The definition of altruism that we employ here is behavior that reduces an individual’s own payoff but raises the group’s overall payoff, which is the effect of a contribution in the Public Good game. This behavior is often described as cooperativeness rather than altruism. In the Social Value Orientation literature (see for example Murphy et al., 2011) there is a clear distinction drawn between Altruism (maximizing the payoff of other individuals) and Cooperativeness (maximizing the income of the group). Our notion of altruism corresponds to the latter.

11 We conducted Chi-squared tests comparing the distribution of the four categories: Free-riders, Altruists, Conditional cooperators, and Others in each pair of treatments. The distributions are significantly different at *p* < 0.01 for all pairs of treatments except for Fear vs. Disgust, which is not significant at *p* < 0.05.

## Appendix A | Experimental Instructions

In this part of the experiment, your earnings will be calculated in ECU (Experimental Currency Units). At the end of the experiment the total amount of ECU you have earned will be converted to Dollars at the following rate:

17ECU=$1

This part of the experiment is divided into 10 periods. All rounds will count for payment. You will be in a group with the 3 other participants.

### Detailed Instructions

At the beginning of each period each participant receives 20 ECU. In the following we call this his or her endowment. Your task is to decide how to use your endowment.

You have to decide how many of the 20 ECU you want to:

- Contribute to a project and;
- How many of them to keep for yourself.

We will play this game on the computer. After choosing your contribution you must press the OK button. Once you have done this, your decision can no longer be revised.

Once all members of your group have made their decision, your screen will show you the total amount of ECU contributed to the project by each of the four group members (including your contribution). This screen shows you how many ECU you have earned.

Your income consists of two parts:

Part (1) The ECU you kept for yourself
Part (2) The income from the project = 50 percent of the total contribution of all 4 group members to the project (including your own contribution).

Your income in ECU in each period:

= Part 1 + Part 2
= (20 - your contribution to the project) + 0.5*(total contributions to the project)

The income of each group member is calculated in the same way, this means that each group member receives the same income from the project.

**Income part (1) The ECU you kept for yourself**

For each ECU that you keep for yourself you earn an income of 1 ECU.

**Income part (2) The income from the project**

For every ECU you contribute to the project instead, the total contribution rises by one ECU. Your income from the project would rise by 0.5*1 = 0.5 ECU. However, the income of the other group members would also rise by 0.5 ECU each, so that the total income of the group from the project would rise by 2 ECU.

Your contribution to the project therefore also raises the income of the other group members. On the other hand you earn an income for each ECU contributed by the other members to the project. For each ECU contributed by any member of the group you earn 0.5*1 = 0.5 ECU.

For example, suppose the total of the contributions of all group members is 60 ECU. In this case each member of the group receives an income from the project of 0.5*60 = 30 ECU.

To check your understanding of the experiment, please answer the following questions:

(1) Suppose each group member has an endowment of 20 ECU. Nobody (including yourself) contributes any ECU to the project. How high is:
  (a) Your income for the period? _________
  (b) The income for each of the other group members for the period? _________
(2) Suppose each group member has an endowment of 20 ECU. You contribute 20 ECU to the project. All other group members contribute 20 ECU to the project.
  (a) What is your income for the period? _________
  (b) The income for each of the other group members for the period? _________
(3) Suppose each group member has an endowment of 20 ECU. The other three group members contribute a total of 30 ECU to the project.
  (a) What is your income if you contribute 0 ECU to the project? _________
  (b) What is your income if you contribute 15 ECU to the project? _________
(4) Suppose each group member has an endowment of 20 ECU. You contribute 8 ECU to the project.
  (a) What is your income if the other group members together contribute a total of 7 ECU to the project? _________
  (b) What is your income if the other group members together contribute a total of 22 ECU to the project? _________

## Appendix B | Manipulation check

In this appendix we report the results from three different manipulation checks of the videos we used to induce emotions. In separate sessions from those of the main study described above, subjects viewed one of the four videos used in this study. In the first two manipulation checks, they subsequently reported the strength, on a scale of 1 - 5, that they felt each of the following emotions indicated on the form shown in Figure B1. The questionnaire items are drawn from the PANAS-X survey (Watson and Clark, 1994). The subjects were undergraduate students at the University of Arizona, the same pool of participants that did the main study reported in the paper. The study was conducted in October and November, 2021.

From the items in the above questionnaire, the following indices were constructed:

Joviality = Average (Cheerful, Joyful, Happy, Excited, Enthusiastic, Energetic)

Fear = Average (Afraid, Frightened, Nervous, Scared)

Hostility = Average (Angry, Irritable, Disgusted, Hostile)

Sadness = Average (Lonely, Downhearted, Sad, Alone)

Attentiveness = Average (Determined, Alert, Attentive, Concentrating).

In the first manipulation check study, there were 48 participants, and 16 viewed each video. They completed the questionnaire above both before and after viewing the video. The average value and standard deviation of each index after viewing each video is reported in Table B1. The table shows that the Happy video significantly increases the level of joviality, while lowering the amount of fear and hostility the average person reports. The Fear video increases the reported level of fear without significantly affecting any of the other four indices. The Neutral video does not increase any of the emotions, though it lowers both hostility and attentiveness.

**TABLE B1:** Average value of emotional indices before and after Happy, Neutral, and Fear videos, manipulation check #1.

Video		Index				
		Joviality	Fear	Hostility	Sadness	Attentiveness
Neutral (*N* = 16)	Before Video	2.84 (0.95)	1.45 (0.66)	1.47 (0.59)	1.47 (0.54)	3.59 (1.02)
	After Video	2.80 (1.08)	1.36 (0.56)	1.17* (0.24)	1.42 (0.60)	3.14** (1.23)
Happy (*N* = 16)	Before Video	2.75 (0.97)	1.66 (0.74)	1.39 (0.54)	1.52 (0.41)	3.88 (0.81)
	After Video	3.35** (0.84)	1.28** (0.40)	1.02** (0.06)	1.34 (0.43)	3.48* (0.71)
Fear (*N* = 16)	Before Video	2.58 (0.89)	1.63 (0.60)	1.38 (0.47)	1.44 (0.66)	3.34 (0.86)
	After Video	2.54 (1.07)	2.15* (1.08)	1.41 (0.60)	1.42 (0.66)	2.98 (1.16)

**: value of index significantly different before and after viewing the video at p < 0.05 according to t-test. **: before and after significantly different at p < 0.01.*

The second manipulation check study had 108 participants and was conducted in September – November 2021. Participants each viewed one of the three videos and completed the questionnaire shown in Figure B1 afterwards. The average responses are shown in Table B2. The superscript *a* indicates that the average of the emotional index was significantly different after viewing the indicated video than the other two videos at *p* < 0.05. The superscripts *b, c, and d* indicates that the average value of the index after the video is significantly different from after exactly one of the other two videos.

**TABLE B2:** Average value of emotional indices after Neutral, Happy, and Fear videos, manipulation check #2.

Video		Index				
		Joviality	Fear	Hostility	Sadness	Attentiveness
Neutral N = 40	After Video	2.92^a^ (0.95)	1.40^d^ (0.81)	1.21 (0.53)	1.39 (0.50)	2.95 (0.92)
Happy N = 35	After Video	3.27^a^ (1.06)	1.23^d^ (0.31)	1.16 (0.30)	1.43 (0.56)	3.12 (1.05)
Fear N = 33	After Video	2.47^a^ (1.07)	2.63^a^ (1.11)	1.48^c^ (0.59)	1.85^a^ (1.07)	3.29 (0.83)

*a: significantly different from both other videos. b: significantly different from Neutral video only. c: significantly different from Happy video only. d: significantly different from Fear video only. All significance thresholds are p < 0.05.*

The data in Table B2 shows that the Happy video generates a higher degree of Joviality than the Neutral treatment or the Fear treatment, but there are no significant differences in the other indices other than leading to lower fear than the Fear video. The Fear video has significantly higher fear than the other two treatments, though it also leads to greater sadness than the other two videos. The only two effects that are consistent over both manipulation checks 1 and 2 are that the Happy video increases Happiness and the Fear video leads to greater fear.

The third manipulation check covers the Disgust and the Neutral videos. In an earlier study, Kugler et al. (2020), reported the results of a manipulation check of the same videos that we used to induce neutrality and disgust with 25 members of the same subject pool that was employed in our study, undergraduate students at the University of Arizona. The PANAS-X protocol was used to measure emotional states both before and after the Neutrality and the Disgust videos. Table B3 below reports the average values of the indices given above for Joviality, Fear, Hostility and Sadness, as well as for Disgust (which was not measured in the data provided above).

**TABLE B3:** Average value of emotional indices before and after Neutral and Disgust videos, manipulation check #3.

Video	Index				
	Happiness	Fear	Anger	Disgust	Sadness
Before any video N = 25	2.52^d^ (0.59)	1.62^a^ (0.69)	1.37^a^ (0.46)	1.08^d^ (0.12)	1.65^a^ (0.72)
Neutral N = 14	2.75^d^ (0.54)	1.28^a^ (0.34)	1.13^a^ (.21)	1.09^d^ (0.15)	1.23^b^ (0.25)
Disgust N = 11	1.59^a^ (0.78)	1.82^a^ (0.77)	1.84^a^ (0.91)	3.27^a^ (1.25)	1.32^b^ (0.39)

*a: significantly different from both other conditions. b: significantly different from the level before video is shown only. c: significantly different from Neutral video only. d: significantly different from Disgust video only. All significance thresholds are p < 0.05.*

Comparison of the data before any video is shown and the after the neutral video is viewed reveals the following pattern. The neutral video yields an emotional state that is similar to that present before the video with regard to Disgust and Happiness, but it lowers Fear, Anger, and Sadness. The Disgust video has a significantly higher level of disgust than before any video is shown, but does not change any other emotion significantly. The Neutral and Disgust videos yield different levels of Happiness, Fear, Anger and Disgust from each other, with the largest being the difference in Disgust.

* Nguyen, Department of Economics, Faculty of Economics and Business, Tilburg University, Tilburg, The Netherlands. Noussair: Department of Economics, Eller College of Management, University of Arizona, Tucson, AZ, United States. Correspondence to cnoussair@arizona.edu. We thank Monica Capra, Jason Shachat, and participants at the 2019 ANZWEE conference at Monash University and at the 2019 Lima Conference on Experimental and Behavioral Economics, at the Catholic University in Lima, Peru, for valuable comments.
